# Polyphenolic Compounds in Extracts from Roasted Grapevine Canes: An Investigation for a Circular Approach to Increase Sustainability in the Viticulture Sectors

**DOI:** 10.3390/molecules28227676

**Published:** 2023-11-20

**Authors:** Veronica D’Eusanio, Lorenzo Morelli, Andrea Marchetti, Lorenzo Tassi

**Affiliations:** 1 Department of Chemical and Geological Sciences, University of Modena and Reggio Emilia, 41121 Modena, Italy; lollo2200@gmail.com (L.M.); andrea.marchetti@unimore.it (A.M.); 2 National Interuniversity Consortium of Materials Science and Technology (INSTM), 50121 Florence, Italy; 3 Interdepartmental Research Center BIOGEST-SITEIA, University of Modena and Reggio Emilia, 42121 Reggio Emilia, Italy

**Keywords:** *Vitis vinifera*, sustainability, *trans*-resveratrol, total phenolic content, HPLC-DAD, UHPLC-MS/MS, waste valorization

## Abstract

In this study, we compared the polyphenolic composition of the roasted grapevine wood chips of four *Vitis vinifera* cultivars—namely, Sorbara, Grasparossa, Malbo Gentile, and Spergola. These waste byproducts have the potential as infusion chips for the aging of alcoholic beverages and vinegars, contributing to an enriched sensory profile. Roasting amplifies aromatic nuances and triggers the depletion of crucial bioactive compounds, including polyphenols. We investigated the extent of polyphenolic loss in the ethanolic extract of roasted grapevine chips to repurpose this waste byproduct and assess its potential. We assessed the levels of *trans*-resveratrol, *trans*-ε-viniferin, *trans*-piceatannol, and the main resveratrol trimer. Our findings indicated a significant decrease in polyphenol content as the roasting temperature increased, from 16.85–21.12 mg GAE/g for grapevine chips roasted at 120 °C to 3.10–7.77 mg GAE/g for those roasted at 240 °C. This study also highlights notable genotypic differences in polyphenolic content. Among the red grape cultivars analyzed, Sorbara exhibited the highest levels (7.77–21.12 mg/GAEg), whereas the white grape cultivar Spergola showed the lowest polyphenolic content (3.10–16.85 mg/GAEg). These findings not only contribute to the scientific understanding of polyphenol stability but also hold practical implications for the enhancement of aged beverages, as well as advancing sustainable practices in the viticulture industries.

## 1. Introduction

The wine industry, deeply rooted in history, culture, and traditions, has long been a hallmark of European excellence [1,2]. Europe boasts the largest winery production and vineyard area in the world and is home to some of the world’s most significant and celebrated wine regions and vintages. Among the many countries that have embraced this ancient craft, Italy, France, and Spain stand as true vanguards, exemplifying the art of wine making on a global scale [3]. This industry not only reflects their history and traditions but also plays a pivotal role in shaping their modern economies, since the export of premium wines to global markets is a key factor in maintaining their commercial balance [3,4]. In this context, Italy is the first largest wine producer, with 49.8 million hectoliters produced in 2022, accounting for 19.3% of the total global wine production. A substantial portion of Italian production is exported, generating a turnover of 7.8 billion euros in 2022. While the wine industry often focuses on the final product, it is important to acknowledge the waste generated throughout the production cycle [5], particularly in countries where a high volume of wine production results in substantial quantities of waste. Various waste streams are generated from grape processing to bottling, including grape skins, stems, lees, and canes [6,7]. Waste disposal practices, including incineration and landfilling, have raised environmental concerns because of their impact on air quality and soil contamination [8]. The shift towards sustainability, promoted by Agenda 2030 for Sustainable Development and crucial to combating climate change, has prompted the industry to reconsider its waste management strategies [9,10,11]. Innovative approaches, such as composting and anaerobic digestion, have proven successful in managing organic waste, yielding valuable byproducts such as nutrient-rich compost and biogas [12,13,14]. The repurposing of grape waste into food products, cosmetics, and pharmaceuticals is being explored, contributing to a circular economy approach [15]. The wine industry’s journey towards sustainable waste management is an integral part of its broader sustainability agenda. Collaborative efforts among wineries, waste management companies, and researchers are crucial for developing and implementing effective waste management strategies. Embracing the principles of the circular economy, in which waste is minimized, resources are optimized, and value is retained, will play a pivotal role in mitigating waste-related challenges in the industry.

Grapevine prunings, which are abundant in vineyards after the annual pruning cycle, present a unique composition that is rich in aromatic compounds, polyphenols, and essential minerals [16,17,18,19]. These compounds, responsible for the flavors and aromas of wines, play a key role in the transformative potential of grapevine prunings. The most important phenolic compounds in grapevine canes are resveratrol and its oligomers, such as *trans-*ε-viniferin and miyabenol C [20,21,22,23]. These polyphenols are known for their antioxidant properties and potential health benefits, such as cancer prevention, reduction in cardiovascular risk, and anti-inflammatory properties. Resveratrol oligomers are characterized by the polymerization of two to eight resveratrol units and comprise the largest group of oligomeric stilbenes [22,23]. *Trans-*ε-viniferin, the main resveratrol dimer, has attracted attention as a phytoalexin and has been reported to exhibit antioxidant, anticancer, antifungal, antibacterial, and antiviral activities [24,25,26,27].

Incineration of grapevine branches and canes is a common practice for managing waste and generating energy, a method that causes air pollution, greenhouse gas emissions, and the loss of valuable organic matter. Moreover, the open burning of agricultural residues has been strictly limited or prohibited by local authorities. For example, in Italy, such practices have been restricted since 2010, as outlined in Legislative Decree D.Lgs. 205/2010 [28,29]. Therefore, it is important to explore new solutions to enhance these waste materials. A proposal already explored in previous studies concerns the use of vine prunings as chips for infusion during the aging process to produce alcoholic beverages and vinegars [17,19]. By carefully processing and preparing prunings as chips for infusion, we can leverage their inherent qualities to enhance the complexity and character of alcoholic beverages, such as wines, brandies, and whiskeys, and elevate the profiles of vinegars. The concept of using wood chips for flavor enhancement during the aging of alcoholic beverages is not new [30,31,32,33]; however, the use of grapevine prunings brings an interesting twist. Drawing from the grapevine’s genetic heritage and regional terroir, infusion with grapevine wood chips holds the promise of imparting distinctive nuances reminiscent of the grape varietals and the environment in which they were cultivated. This technique can provide an additional layer of complexity in the aging process, enabling beverage producers to craft unique, high-quality products that resonate with both connoisseurs and enthusiasts. 

A crucial aspect of harnessing grapevine prunings as chips for infusion is the roasting phase, a process that plays an important role in enriching the aromatic profile of wood and further refining the nuances imparted to aged beverages [34]. Studying the dynamics of polyphenol content variation at different roasting temperatures is essential. The interplay between the temperature and the resulting chemical reactions is a critical focal point, allowing us to determine the optimal conditions for extracting and preserving these valuable compounds. Understanding this intricate relationship will enable us to fine-tune the sensory attributes of the final products while maximizing the potential health-enhancing benefits of grapevine wood infusion.

In this study, we focused our investigation on the grapevine prunings of four distinct cultivars native to the Modena–Reggio Emilia district: Malbo Gentile (MG), Sorbara (SO), Grasparossa (GR), and Spergola (SP). This selection encompasses three red grape Lambrusco cultivars (MG, SO, and GR) and one white grape cultivar (SP), presenting an opportunity to explore both intragenotypic and intergenotypic differences in polyphenolic content following roasting. The inclusion of both red and white grape cultivars is particularly intriguing, as it allows us to assess how varietal characteristics and the presence of anthocyanins, predominantly found in red grape varieties, may influence the phenolic composition after roasting. 

In this paper, we explore the scientific context behind the utilization of grapevine prunings as chips for infusion, discuss the extraction of polyphenols, the interplay between roasting temperature and the preservation of phenolic compounds, and the potential benefits for the final products. Understanding these genotypic variations is of paramount importance, not only for fine-tuning infusion techniques, but also for broader viticultural and winemaking practices. By investigating the complex connection between grape variety, roasting temperature, and resulting polyphenol profiles, we can pave the way for a more tailored approach in crafting beverages that not only reflects the unique terroir of the Modena–Reggio Emilia district but also captures the essence of each cultivar. The grapevine chips from the four cultivars were roasted at different temperatures (120, 140, 160, 180, 200, 220 and 240 °C), and polyphenols were extracted with ethanol. The total phenolic content and quantification of *trans*-resveratrol, *trans*-ε-viniferin, *trans*-piceatannol, and the main resveratrol trimer were determined. 

Through this research, we aim to contribute to the advancement of innovative and sustainable practices in the beverage and wine industry while also shedding light on the broader concept of reimagining waste materials as valuable resources. 

## 2. Results and Discussion

**Figure 1:**
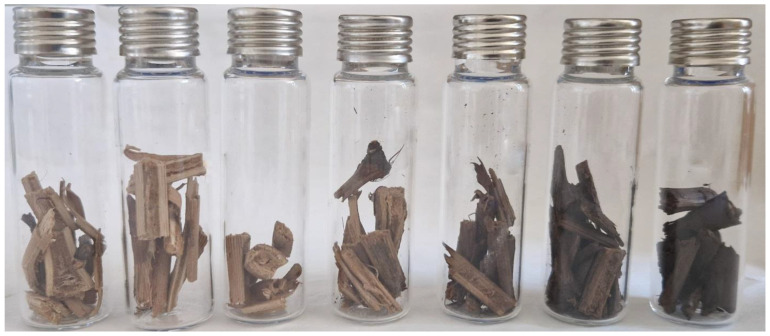
Grapevine chips from the Lambrusco Sorbara cultivar roasted at different temperatures (from left to right: 120, 140, 160, 180, 200, 220 and 240 °C).

Enhancing the wood’s aromatic profile with unique and characteristic notes is the key aim of the roasting process. These notes depend on the analyzed cultivar, as demonstrated in prior studies that employ HS-SPME-GC-MS analysis [35]. Thermally activated reactions generate a broad spectrum of volatile organic compounds (VOCs), the quantity and prevalence of which vary significantly with changes in roasting temperature [36,37]. At higher temperatures, the contributions linked to the degradation of cellulose, hemicellulose, and lignin biopolymers become more pronounced. Conversely, at lower temperatures, thermoactivated reactions, such as the Maillard reaction, take place [38,39]. These processes not only entail substantial modification of the wood structure but also result in a shift in the wood color, progressively darkening, as can be seen in Figure 1.

To conduct a thorough investigation encompassing the four grapevine cultivars, meticulous sampling methods were used to ensure the acquisition of highly representative samples. The work plan involved the following steps for sampling and analyzing grapevine cane samples from the Sorbara, Grasparossa, Spergola, and Malbo Gentile cultivars. First, grapevine plants were sampled after grape harvest, with a total of 20 plants selected for each cultivar. The collected grapevine wood amounted to approximately 6 kg. All collected samples were completely intact, with no signs of damage caused by fungal attacks or other pathologies. Subsequently, the grapevine cane samples were debarked and subjected to roasting at eight different temperatures ranging from 120 to 260 °C. For each roasting temperature, four replicates were performed to ensure the reliability of the results. The selection of eight temperatures was based on the need to capture a comprehensive spectrum of thermal conditions during the roasting process. This range was chosen to encompass a variety of temperatures relevant to the transformation of wood following heat treatment. Finally, the roasted grapevine chips underwent sonication and macerative solvent extraction in ethanol at 80 °C. This process was repeated four times in order to obtain four replicates. 

The process of removing the outer bark from the specimens is designed to mitigate the presence of pesticides and contaminants. Additionally, strict adherence to Regulation (EC) No. 1107/2009 of the European Parliament and of the Council [40,41], dated 21 October 2009, mandates precise compliance with safety intervals between the final pesticide application and fruit harvesting. Collecting vine shoots post-harvest thus ensures a high level of phytosanitary safety. 

### 2.1. Proximate Analysis

Table 1 lists the proximate chemical composition of the grapevine chips from the four studied cultivars: three PGI (Protected Geographical Indication)–PDO (Protected Designation of Origin) Lambrusco species, namely Malbo Gentile, Sorbara, and Grasparossa, and PDO Spergola. Plant matrices exhibit chemical compositions and physical properties that significantly hinge on their geographical origin, maturation degree [19,42], genotypic characteristics [18,19,43], the soil type of cultivation, and meteorological conditions. The sampling process was systematically carried out within the same geographical area and during a consistent period of the year (20–25 September 2022). This approach ensured constant exposure to atmospheric conditions, as well as uniform soil characteristics and vine plant maturation levels across samples. Therefore, the variations highlighted in Table 1 primarily stem from genotypic differences.

The moisture content was similar among the woods of the red grape cultivars (Malbo Gentile, Sorbara, and Grasparossa), while a slightly higher value characterized the white grape variety Spergola. This observation is in agreement with earlier studies [44]. Woody matrices, primarily consisting of hemicelluloses, cellulose, and lignin [45,46,47], typically exhibit elevated levels of carbon and oxygen (C% and O%, respectively), along with lower levels of nitrogen and sulfur (N% and S%, respectively). The findings presented in Table 1 align with the established literature, where grapevine wood is characterized by approximately 40–42% cellulose, 24–26% hemicellulose, and 18–21% lignin [48]. The residual portion comprises minor compounds, including lipids, essential minerals, aromatic compounds, and bioactive molecules, such as polyphenols, vitamins, and other antioxidants [16,17,49,50,51]. This extractable component fraction accounts for 8–10%. 

### 2.2. Effect of Roasting on Sample Mass Loss

Table 2 shows the mass loss (Δm%) values obtained from the roasting at different temperatures of grapevine cane samples of the four *Vitis vinifera* cultivars Malbo Gentile (MG), Sorbara (SO), Grasparossa (GR), and Spergola (SP). Values are expressed as mean ± standard deviation of four replicates. Data followed by different letters in a column indicate statistically significant differences (*p* < 0.05).

The data obtained are consistent with those reported in previous studies [17,19]. To obtain a comprehensive understanding of the changes that roasting temperature induces in the composition of matrices, it is important to look at the mass losses, which enable a quick assessment of the degree of change resulting from heat treatment. The observed trend was almost the same for all the samples examined, with a progressive decrease in the mass up to 220 °C, followed by a more marked decrease at higher temperatures. In absolute terms, the highest mass losses were observed for the GR and MG cultivars, which showed a loss of 56.23–58.91% at 260 °C. In contrast, SP and SO showed a mass loss of 42–46%, indicating a probable higher heat resistance, rigidity, and greater wood density for these cultivars. Even if the greatest mass loss was observed above the roasting temperature of 220 °C, the first degradation mechanism of hemicellulose and cellulose occurred already from 160 °C, which consists of the loss of intermolecular water [36]. At this temperature, the effect of roasting became significant because the wood gradually stiffened, losing its elasticity, and the loss of structural water began. The slow increase in mass loss up to 140 °C is due to moisture removal and the loss of volatile organic compounds (VOCs) and semi-volatile organic compounds (SVOCs). Proteins persist up to 200–220 °C; therefore, starting from this temperature range, the chemical structure of the biomass begins to destabilize and partly depolymerize [52,53]. Furthermore, above 200 °C, hemicelluloses and cellulose start to decompose effectively. The structural decay reactions of proteins occur at temperatures up to 240 °C, leading to a strong mass loss above this temperature. The degradation of lignin occurs over a wide thermal range, from 150 °C to over 500 °C, because of its high three-dimensional complexity. The units of lignin vary in composition and possess different levels of thermal stability [54].

### 2.3. TPC–Total Phenolic Content

Table 3 shows the total phenolic content (TPC), obtained through the Folin-Ciocalteau method, of the ethanolic extracts from roasted grapevine chips.

Upon analyzing the data presented in Table 3, several key insights regarding the influence of roasting temperature on the total phenolic content (TPC) of grapevine wood chips were obtained. The TPC values exhibited notable variations in the direct response to the temperature at which the chips were roasted. This observation aligns with the well-established fact that polyphenols, being thermally labile molecules, undergo structural alterations under the influence of heat [55]. The same trend was observed for the four cultivars in which an increase in roasting temperature was consistently associated with a decrease in TPC. Examining the variations within individual cultivars emphasizes the intricate relationship between grape variety and roasting temperature. Notably, among the red grape cultivars, Lambrusco Sorbara demonstrated consistently higher TPC values at all temperatures. This observation highlights the specific composition and phenolic stability of Sorbara grapevine prunings, possibly due to varietal-specific chemical structures that influence wood thermal stability. The samples from the Spergola cultivar, the only white grape variety, consistently exhibited the lowest TPC values at all the roasting temperatures tested. Red grape varieties possess higher levels of anthocyanins and proanthocyanidins, which significantly contribute to their phenolic content. Anthocyanins, which are responsible for the characteristic color of red grapes, also exhibit greater thermal stability than other polyphenols. Therefore, during roasting, red grape-derived prunings may retain a higher proportion of phenolic compounds, resulting in elevated TPC values. Factors such as chemical structure, the presence of certain functional groups, interactions between phenolic compounds and matrix constituents, and environmental factors such as pH can collectively influence the susceptibility of polyphenols to thermal degradation [56].

### 2.4. Stilbenoid Concentration in the Different Samples of Roasted Grapevine Chips

This study investigates four main stilbenoids: *trans*-resveratrol, *trans*-ε-viniferin, *trans*-piceatannol, and the main trimer of *trans*-resveratrol. The latter, identified as miyabenol C or one of its various isomers [57], exhibits a molecular ion [M − H]^−^ of 679 amu. The detected stilbenoids were effectively quantified, and the total stilbenoid content was represented as the sum of their concentrations expressed in *trans*-resveratrol equivalents. 

**Figure 2:**
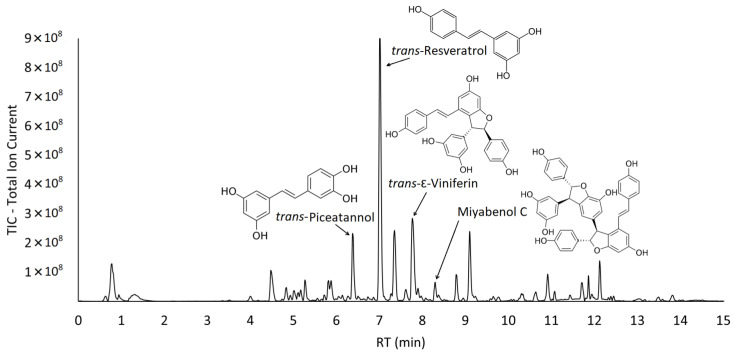
UHPLC-MS/MS chromatogram of the ethanolic extract of the sample SO160; chemical structure of the identified stilbenoids (*trans*-piceatannol, *trans*-resveratrol, *trans*-ε-viniferin, miyabenol C as the main trimer of *trans*-resveratrol).

Figure 2 shows a representative chromatogram of the reversed-phase UHPLC-MS/MS analysis of the ethanolic extract of SO160.

As can be observed in Figure 2, the signals present in the chromatogram are not exclusively associated with stilbenoids. They may encompass phenolic acids, flavonoids, and organic acids—compounds not specifically investigated in this study but previously identified by other authors [58]. 

Table 4 presents the results of the UHPLC-MS/MS analysis. Data are expressed as mean ± standard deviation of four replicates. Data followed by different letters in a column indicate statistically significant differences (*p* < 0.05).

**Figure 3:**
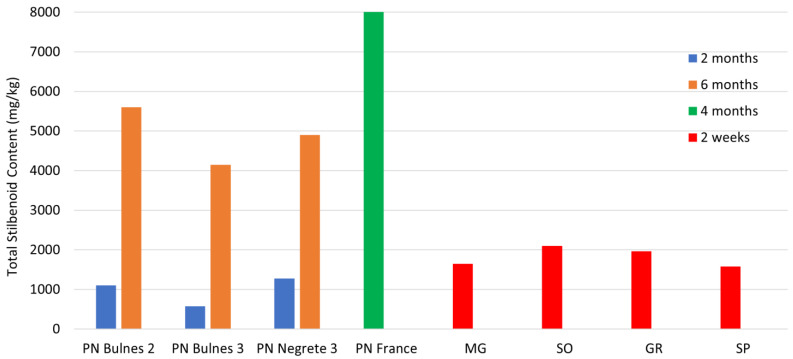
Comparison of the data related to the total stilbenoid content among the examined samples (MG, SO, GR, SP) and some literature data [21,42]. The different colors indicate the maturation times for the grapevine wood samples.

Before examining the values obtained in this work, let us focus on the data related to the stilbenoid content of the untreated natural samples. It is well known from the literature that plant enzymatic systems remain active for at least 6–8 months, even after the removal of pruning shoots, resulting in an increase in the concentration of stilbenoids produced and synthesized by natural biocycles [21]. Therefore, we collected some literature data for an effective comparison with our results (Figure 3) [21,42]. The data examined relate to the different maturation times for grapevine wood samples. In particular, the PN Bulnes 2, PN Bulnes 3, and PN Negrete cultivars were analyzed after 2 and 6 months from collection, while the PN France cultivar was analyzed after 4 months. It is important to note that our samples were analyzed only 2 weeks after collection. 

In Figure 3, the trend related to the stilbenoid content in grapevine shoots from different Vitis vinifera cultivars from various geographical regions is depicted.

To simplify the comparison, we present data for only one variety, namely Pinot Noir (PN) from Bulnes and Negrete (Chile), as well as Villenave d’Ornon (France). The plant materials underwent various stages of maturation: the Chilean PN samples were matured for 2 and 6 months after harvest before analysis, while the French samples were from shoots matured for 4 months. It is interesting to note that the Chilean samples showed an approximately fivefold increase in stilbenoid content from 2 to 6 months of maturation, while the values for the French samples reached approximately 8 g/kg of dry wood after 4 months of storage.

Our samples, originating from cultivars different from PN, were analyzed approximately 2 weeks after harvest. However, the stilbenoid content is approximately twice that of the Chilean PN samples aged for 2 months and roughly half of the values for shoots aged for 6 months.

The data for the French PN averages are significantly higher than our values by a factor ranging from 4 to 5 times. This substantial difference can be attributed, in part, to the different cultivars being compared, but especially to the wood aging time, 4 months versus 2 weeks. These quantitative observations should be taken into careful consideration if there is a need to utilize vine pruning waste biomass to extract bioactive molecules and compounds for productive purposes, starting from mature wood under ordinary conditions.

The significant differences in stilbenoid concentrations among the samples from different grape cultivars, despite identical cultivation under consistent climatic and geographical conditions, highlight the presence of strong genotypic differences. Spergola, the only white-berry cultivar, had the lowest overall stilbenoid content. Moreover, the most significant loss (−90%) was observed when moving from the 120 °C-roasted sample to the 240 °C-roasted one. In contrast, the other three cultivars showed a reduction of approximately 85%. 

*Trans*-resveratrol had the highest concentration, followed by *trans*-ε-viniferin, *trans*-piceatannol, and the main trimer of *trans*-resveratrol. As expected, the concentration values decreased as the roasting temperature increased, mainly because of the thermal instability of the stilbenoids. This leads to the non-quantification of *trans*-piceatannol and the main trimer of *trans*-resveratrol in some samples roasted at least 220 °C. 

**Figure 4:**
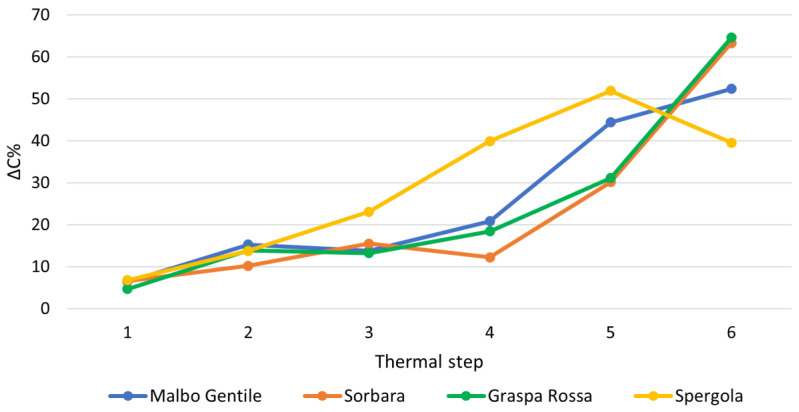
Relative percentage difference in the total concentration of stilbenoids between two consecutive thermal steps. Thermal steps: 1 = 120–140 °C; 2 = 140–160 °C; 3 = 160–180 °C; 4 = 180–200 °C; 5 = 200–220 °C; 6 = 220–240 °C.

Figure 4 illustrates the relative percentage difference in the total concentration of stilbenoid between two consecutive thermal steps. ΔC% is calculated as:

$$∆C\%=\frac{C_{i}-C_{f}}{C_{i}}\%$$

where C_i_ and C_f_ represent the total stilbenoid concentrations in two consecutive thermal steps (for example, the total stilbenoid concentrations in MG120 and MG140). This value provides information on the extent to which a specific thermal step affects the total stilbenoid concentration.

From Figure 4, it is evident that an increase in temperature not only results in the reduction of stilbenoids, as indicated in Table 4, but also amplifies the degree of the reduction itself. This effect is particularly pronounced in the Lambrusco Malbo Gentile and especially Spergola cultivars, which experience a loss exceeding 40% during the 200–220 °C thermal step. This may deter the use of roasting temperatures above 200 °C to retain a large quantity of stilbenoids. Moreover, a recent study demonstrated that temperatures exceeding 200 °C can result in the emission of certain toxic compounds, including benzene, toluene, and xylenes [35]. Conversely, starting at 160 °C, volatile organic compounds characteristic of wood roasting are emitted, contributing to a pleasant aroma. Therefore, selecting temperatures within the range of 160–180 °C can constitute a favorable compromise between preserving the total stilbenoid content and enhancing the aroma imparted by roasted grapevine chips.

To the best of our knowledge, according to studies concerning the concentration of stilbenes in grapevine canes, it has been observed that the concentration of *trans*-resveratrol falls within the range of 100 to 4700 mg/kg dw, while *trans*-ε-viniferin ranges from less than 100 to 1700 mg/kg dw. It is important to note that these studies pertain to grapevine chips that have not undergone any thermal treatment. Our findings are almost in line with the data available in this dataset, indicating the reliability and consistency of our results. There were exceptions to samples MG240, SP220, and SP240, wherein the concentration of *trans*-resveratrol fell below the lower limit of the range (100 mg/kg dw). 

The levels of stilbenes in our samples indicate substantial potential for commercial applications due to their strong antioxidant properties. This observation becomes even more interesting when considering that it involves waste grapevine wood, traditionally perceived as having little inherent value. By highlighting the modest yet existent levels of stilbenes, we underscore the possibility of extracting value from what might otherwise be overlooked as mere waste material. Stilbenes, such as *trans*-resveratrol and *trans*-ε-viniferin, are highly regarded for their bioactive properties, making them valuable in both the nutraceutical and pharmaceutical industries. Additionally, it is worth highlighting that stilbenes are known for their significant anti-phytopathogenic properties, making them advantageous for plant protection. 

## 3. Materials and Methods

### 3.1. Reagents and Standards

Ethanol (96%) was obtained from Carlo Erba Reagents, Milano (Italy). HPLC-grade methanol and formic acid were obtained from Sigma Aldrich (St. Luis, MO, USA). Deionized water was produced using a Milli-Q Plus (Millipore system). *Trans*-resveratrol standard was obtained from Sigma Aldrich, distributed by Merck KGaA (Darmstadt, Germany).

### 3.2. Sample Preparation

Grapevine canes of *Vitis vinifera* cv. Malbo Gentile (MG), Sorbara (SO), Grasparossa (GR) and Spergola (SP) were collected in a farm of the territorial district of Modena (Italy). The vine plants grew under the same climatic and hydric conditions and on the same soil. To avoid any contamination by pesticides and fungicides typically deposited on the outer bark, we selected only the internal parts of the grapevine canes. The outer layer was peeled off and manually removed. 

The sampling was carried out in September 2022, and the pruned canes were air-dried at room temperature for one week. 

All the samples were manually dehulled, minced, reduced to 4–5 mm chips, closed in glass containers, and thermally treated for 2 h at different temperatures: 120, 140, 160, 180, 200, 220, 240 °C in an inert atmosphere (N_2_). They were then manually ground into powder and extracted through maceration with ethanol. 

### 3.3. Proximate Composition

Moisture, ash, and elemental analysis were determined following the methods recommended by the Association of Official Analytical Chemists [59]. Moisture content was determined by drying the grapevine cane samples at 105 °C to a constant weight. The ash content was determined using a laboratory furnace at 550 °C and the temperature was gradually increased. Each measurement was performed in quadruplicate, and the results were averaged.

### 3.4. Macerative Solvent Extraction

For the extraction of the active components, 2 g (±1%) of milled grapevine canes was placed into the extraction vessel, covered with ethanol 96% *v*/*v* (10 mL/g of powdered material), and closed with a PBT screw cap. They were sonicated for 1 h at 35 °C and macerated for 24 h in an oven at 80 °C. The extracts were then filtered, and the solid was washed three times with 10 mL of ethanol to collect the washing in the filtrate. The extracts were dried using a rotary evaporator, and the final step was conducted in an oven at 70 °C for 2 h before quantification. The final products had a pitchy/powdery consistency and were stored at 4 °C until analysis. 

### 3.5. HPLC-DAD Analysis

The quantification of stilbenes was performed using HPLC-DAD analysis. *Trans*-resveratrol standards were processed and analyzed in replicates. Analytes were separated using a C18 Cortecs column (2.7 μm, 2.1 mm × 100 mm) (Waters Co., Milford, MA, USA). The analysis was performed at a constant flow rate of 0.3 mL/min, using a binary gradient elution with acidified water (0.1% formic acid), methanol 80:20 (mobile phase A), and methanol 100% (mobile phase B). The gradient was programmed as follows: 0 min, 20% B; 25 min, 98%, and held for 5 min to return to the initial conditions. Finally, a conditioning cycle of 10 min under initial conditions was adopted. UV detection was performed at 280 nm and 325 nm. The stilbenoid content was determined from the calibration curve of the *trans*-resveratrol standards (injected concentrations ranging from 2 to 500 μg/mL). The linearity of the response (correlation coefficient R^2^ = 0.9982) of the *trans*-resveratrol standards was determined by plotting the peak area versus its concentration. 

### 3.6. UHPLC-MS/MS Analysis

A UHPLC system coupled with an Orbitrap Q-Exactive equipped with a micro-ESI (Thermo Fisher Scientific, Waltham, MA, USA) was used. All samples were processed and analyzed in replicates. Analytes were separated using a C18 Cortecs column (2.7 μm, 2.1 mm × 100 mm) (Waters Co., Milford, MA, USA). The analysis was performed under the same conditions as those used for the quantification of *trans*-resveratrol via HPLC-DAD analysis. The analysis was performed at a constant flow rate of 0.3 mL/min, using a binary gradient elution with acidified water (0.1% formic acid), methanol 80:20 (mobile phase A), and methanol 100% (mobile phase B). The gradient was programmed as follows: 0 min, 20% B; 25 min, 98%, and held for 5 min to return to the initial conditions. Finally, a conditioning cycle of 10 min under initial conditions was adopted. Electrospray ionization in negative mode was used. Detection was performed considering a mass range of 50–1000 u.m.a.

Compounds in the extracts were identified according to their mass spectrum, and for *trans*-resveratrol only, the retention time was compared to the external standard. Quantification was performed using an external calibration curve, with *trans*-resveratrol as the external standard, and the results were expressed as *trans*-resveratrol equivalents.

### 3.7. Statistical Analysis

The experimental data were compared by conducting an analysis of variance (one-way ANOVA) with Tukey-Kramer honestly significant difference (HSD) post hoc testing, by using the Matlab^®^ 2023a environment (Mathworks Inc., Natick, MA, USA). The level of significance was determined at *p* < 0.05 to see whether there were statistical differences between the mean values. 

## 4. Conclusions

In this study, an analysis of the total phenolic content (TPC) and stilbenoid content in roasted grapevine canes derived from three Lambrusco cultivars, Sorbara, Grasparossa and Malbo Gentile, and Spergola cultivar was conducted. Stilbenoids are compounds of significant interest because of their potential applications in the pharmaceutical and nutraceutical sectors. Additionally, these molecules have displayed promising anti-phytopatogenic properties, opening doors for potential applications in agriculture. Our main aim was to demonstrate that grapevine canes maintained substantial concentrations of stilbenoids, even after heat treatment. This resilience offers prospects for applications in the food industry, particularly as wood chips used for infusion during the aging of alcoholic beverages and vinegars. In this context, roasting is fundamental for boosting the aromatic notes of grapevine wood. Furthermore, we sought to investigate whether variances in stilbenoid content exist among different cultivars and are influenced by genotypic variations. The stilbenoids quantified included *trans*-resveratrol, *trans*-viniferin, *trans*-piceatannol, and the main trimer of *trans*-resveratrol. Among the cultivars studied, Spergola exhibited the lowest concentration values. In contrast, the Sorbara cultivar displayed the highest stilbenoid values, followed by GraspaRossa and Malbo Gentile. The TPC followed the same trend among the four cultivars. The stilbenoid content decreased with increasing roasting temperature, and the magnitude of the decrease progressively increased. Notably, the concentration values observed in our roasted grapevine cane samples were consistent with the trends observed in similar non-heat-treated samples. This reaffirms that despite the roasting process, these materials retain their high polyphenol content.

Finally, the findings of this study hold considerable significance in the context of sustainable resource utilization. By demonstrating the retention of high stilbenoid content in roasted grapevine canes, we highlight a sustainable avenue for the reintegration of agri-food byproducts into production cycles. This not only aligns with the principles of a circular economy but also reduces the ecological burden associated with waste disposal.

## Figures and Tables

**Table 1:** Proximate chemical composition of three Lambrusco cultivars Malbo Gentile, Sorbara and Grasparossa, and Spergola grapevine canes.

	Malbo Gentile	Sorbara	Grasparossa	Spergola
Moisture (at 105 °C) %	18.0 ± 0.1	17.7 ± 0.2	17.6 ± 0.1	22.9 ± 0.4
C% *	45.7 ± 0.1	46.8 ± 0.1	46.9 ± 0.3	44.9 ± 0.2
H% *	6.99 ± 0.06	6.94 ± 0.07	6.92 ± 0.05	6.98 ± 0.07
N% *	0.46 ± 0.03	0.46 ± 0.05	0.44 ± 0.03	0.48 ± 0.04
S% *	<0.1	<0.1	<0.1	<0.1
O% * ^#^	43.8 ± 0.08	42.7 ± 0.06	42.6 ± 0.07	44.6 ± 0.05
Ash% *	3.01 ± 0.04	3.08 ± 0.05	3.09 ± 0.06	3.00 ± 0.05

* on dry basis at 105 °C. ^#^ by difference.

**Table 2:** Mass variation of roasted grapevine chips at different temperatures for the four *Vitis vinifera* cultivars. Data followed by different letters in a column indicate statistically significant differences (*p* < 0.05).

−Δm%				
t/°C	Malbo Gentile (MG)	Sorbara (SO)	Grasparossa (GR)	Spergola (SP)
120	29.69 ± 0.26 a	16.88 ± 0.24 a	12.90 ± 0.27 a	16.07 ± 0.19 a
140	36.29 ± 0.33 b	25.16 ± 0.18 b	34.09 ± 0.20 b	24.37 ± 0.23 b
160	37.18 ± 0.23 c	26.96 ± 0.22 c	42.04 ± 0.27 c	28.60 ± 0.19 c
180	37.64 ± 0.30 cd	28.66 ± 0.25 d	42.17 ± 0.25 c	29.46 ± 0.23 d
200	38.25 ± 0.33 d	30.60 ± 0.32 e	43.66 ± 0.27 d	30.60 ± 0.25 e
220	40.47 ± 0.29 e	34.38 ± 0.25 f	47.52 ± 0.34 e	33.42 ± 0.26 f
240	46.26 ± 0.32 f	39.21 ± 0.39 g	53.40 ± 0.35 f	37.30 ± 0.28 g
260	56.23 ± 0.37 g	46.24 ± 0.42 h	58.91 ± 0.50 g	42.72 ± 0.37 h

**Table 3:** TPC (mg GAE/g) of ethanolic extracts from roasted grapevine chips.

TPC (mg GAE */g)				
°C	MG	SO	GR	SP
nts #	21.84 ± 1.22 ^aAB^	23.43 ± 1.36 ^aAB^	24.63 ± 1.25 ^aAB^	19.93 ± 1.15 ^aAB^
120	18.56 ± 0.97 ^aAB^	21.12 ± 1.32 ^aA^	20.01 ± 0.99 ^aA^	16.85 ± 0.87 ^aB^
140	16.35 ± 1.12 ^bAC^	20.31 ± 1.11 ^abB^	17.56 ± 1.21 ^bAB^	14.32 ± 0.96 ^bC^
160	14.25 ± 0.86 ^bcAC^	18.52 ± 0.96 ^bcB^	15.48 ± 1.14 ^bcA^	12.22 ± 0.64 ^cC^
180	12.69 ± 0.89 ^cdA^	17.44 ± 0.71 ^cB^	13.2 ± 0.97 ^cdA^	10.64 ± 0.43 ^cD^
200	10.79 ± 0.46 ^dA^	12.58 ± 0.87 ^dB^	11.1 ± 0.62 ^deAB^	8.62 ± 0.21 ^dC^
220	8.36 ± 0.65 ^eA^	11.43 ± 0.53 ^dB^	9.59 ± 0.33 ^eC^	5.44 ± 0.11 ^eD^
240	4.53 ± 0.11 ^fA^	7.77 ± 0.13 ^eB^	6.35 ± 0.21 ^fC^	3.10 ± 0.09 ^fD^

* GAE = Gallic Acid Equivalent; nts # = no thermal stress; Data are expressed as mean ± standard deviation of four replicates. Means in the same column followed by the same lowercase letter are not significantly different, whereas means in the same row followed by the same uppercase letter are not significantly different. (Lowercase letters for roasting temperature effect; uppercase letters for cultivar effect; Tukey-Kramer HSD test, at *p* < 0.05).

**Table 4:** Stilbenoid concentrations in grape pruning cane samples roasted at different temperatures. Data followed by different letters in a column indicate statistically significant differences (*p* < 0.05).

Stilbenoid Concentration (mg/kg) ^1,2^					
Sample	*trans*-Resveratrol	*trans*-Piceatannol	*trans*-ε-Viniferin	Main Trimer	Total
MG	997.3 ± 160.4 a	213.5 ± 51.8 a	281.2 ± 71.6 a	152.7 ± 57.5 a	1645 ± 345 a
MG120	956.7 ± 166.3 a	201.7 ± 62.3 a	268.1 ± 67.5 a	143.2 ± 66.3 a	1570 ± 372 a
MG140	903.9 ± 160.7 a	185.3 ± 48.5 ab	259.8 ± 61.2 ab	121.3 ± 47.8 a	1470 ± 318 a
MG160	741.3 ± 153.5 ab	156.8 ± 46.2 ab	246.9 ± 63.6 ab	100.6 ± 34.3 ab	1246 ± 298 a
MG180	643.8 ± 141.1 ab	136.8 ± 33.7 ab	223.5 ± 52.9 ab	71.02 ± 20.1 ab	1075 ± 248 ab
MG200	486.2 ± 138.6 bc	110.6 ± 39.9 ab	200.3 ± 49.8 abc	54.36 ± 9.9 b	851.5 ± 238.2 abc
MG220	233.1 ± 100.2 cd	83.51 ± 21.2 b	156.9 ± 47.6 bc	NQ	473.5 ± 169.0 bc
MG240	96.23 ± 31.20 d	NQ	129.3 ± 38.9 c	NQ	225.5 ± 70.1 c
SO	1359 ± 164 a	247.4 ± 72.4 a	321.7 ± 76.8 a	167.6 ± 27.3 a	2095 ± 346 a
SO120	1269 ± 188.3 a	231.8 ± 77.5 a	299.6 ± 88.3 a	155.6 ± 36.9 a	1956 ± 391 a
SO140	1211 ± 182.3 ab	200.6 ± 69.9 a	276.2 ± 49.6 a	139.3 ± 41.0 ab	1827 ± 343 a
SO160	1082 ± 183.6 ab	189.3 ± 56.8 a	259.9 ± 80.7 ab	110.3 ± 25.8 ac	1641 ± 347 ab
SO180	911.3 ± 167.6 abc	169.8 ± 44.1 a	226.3 ± 52.3 ab	79.92 ± 19.9 bd	1387 ± 284 ab
SO200	796.2 ± 151.0 bc	144.8 ± 45.6 a	209.3 ± 47.6 ab	67.31 ± 21.3 cd	1218 ± 265 ab
SO220	523.4 ± 110.2 cd	109.7 ± 36.9 a	177.4 ± 35.5 ab	40.36 ± 7.8 cd	850.9 ± 190.4 bc
SO240	179.3 ± 80.9 d	NQ	133.3 ± 20.1 b	NQ	312.6 ± 101.0 c
GR	1276 ± 188.7 a	231.7 ± 27.2 a	299.3 ± 56.5 a	158.0 ± 30.6 a	1965 ± 307 a
GR120	1220 ± 177.6 a	219.3 ± 20.6 a	284.6 ± 65.8 a	149.7 ± 21.0 a	1874 ± 285 a
GR140	1189 ± 171.2 a	199.6 ± 38.6 ab	270.5 ± 71.9 ab	127.6 ± 33.8 a	1787 ± 315 a
GR160	1002 ± 160.3 ab	177.6 ± 33.9 ab	253.2 ± 75.3 ab	105.9 ± 15.5 ab	1539 ± 285 ab
GR180	897.3 ± 138.7 ab	143.6 ± 41.8 abc	220.9 ± 62.2 ab	74.66 ± 10.2 b	1336 ± 253 ab
GR200	703.6 ± 140.2 bc	122.3 ± 22.4 bc	204.7 ± 48.5 ab	59.32 ± 10.4 b	1090 ± 221 bc
GR220	486.3 ± 120.3 cd	96.3 ± 18.9 c	168.3 ± 33.6 b	NQ	750.9 ± 172.8 cd
GR240	135.6 ± 88.9 d	NQ	130.6 ± 42.5 b	NQ	266.2 ± 131.4 d
SP	960.3 ± 133 a	203.1 ± 54.9 a	272.4 ± 62.5 a	143.2 ± 45.7 a	1579 ± 296 a
SP120	901.7 ± 152.3 a	188.5 ± 49.8 a	255.3 ± 66.9 a	135.6 ± 58.3 a	1481 ± 347 a
SP140	864.2 ± 144.6 a	163.2 ± 33.7 ab	241.9 ± 67.5 a	111.9 ± 46.9 ab	1381 ± 293 a
SP160	710.6 ± 133.6 ab	150.2 ± 38.7 ab	233.6 ± 31.1 ab	96.3 ± 22.1 ab	1191 ± 225 a
SP180	530.5 ± 117.3 bc	129.8 ± 40.2 ab	200.4 ± 77.6 ab	55.51 ± 15.3 ab	916.2 ± 250.4 ab
SP200	261.7 ± 99.8 cd	100.3 ± 35.9 ab	148.3 ± 36.6 ab	40.32 ± 8.8 b	550.6 ± 181.1 bc
SP220	80.24 ± 23.5 d	74.32 ± 20.1 b	110.3 ± 33.7 ab	NQ	264.9 ± 77.3 c
SP240	68.88 ± 34.6 d	NQ	91.32 ± 21.1 b	NQ	160.2 ± 55.7 c

^1^ Data are expressed as mean ± standard deviation of four replicates. ^2^ Data are expressed as *trans*-resveratrol equivalents [18,19]. NQ means detected but not quantified because of very low levels.

## Data Availability

Data are contained within the article.
